# Colonization of the ocean floor by jawless vertebrates across three mass extinctions

**DOI:** 10.1186/s12862-024-02253-y

**Published:** 2024-06-13

**Authors:** Chase Doran Brownstein, Thomas J Near

**Affiliations:** 1 Department of Ecology and Evolutionary Biology, Yale University, New Haven, CT 06511 USA; 2 Yale Peabody Museum, Yale University, New Haven, CT 06511 USA

**Keywords:** Hagfishes, Phylogenetics, Jawless Vertebrates, Continental Slope, Habitat

## Abstract

**Background:**

The deep (> 200 m) ocean floor is often considered to be a refugium of biodiversity; many benthic marine animals appear to share ancient common ancestry with nearshore and terrestrial relatives. Whether this pattern holds for vertebrates is obscured by a poor understanding of the evolutionary history of the oldest marine vertebrate clades. Hagfishes are jawless vertebrates that are either the living sister to all vertebrates or form a clade with lampreys, the only other surviving jawless fishes.

**Results:**

We use the hagfish fossil record and molecular data for all recognized genera to construct a novel hypothesis for hagfish relationships and diversification. We find that crown hagfishes persisted through three mass extinctions after appearing in the Permian ~ 275 Ma, making them one of the oldest living vertebrate lineages. In contrast to most other deep marine vertebrates, we consistently infer a deep origin of continental slope occupation by hagfishes that dates to the Paleozoic.

**Conclusion:**

Our results establish hagfishes as ancient members of demersal continental slope faunas and suggest a prolonged accumulation of deep sea jawless vertebrate biodiversity.

**Supplementary Information:**

The online version contains supplementary material available at 10.1186/s12862-024-02253-y.

## Introduction

The asymmetrical accumulation of ancient biodiversity across ecosystems has fascinated biologists for centuries. Charles Darwin recognized that several ancient clades he dubbed ‘living fossils’ all lived in freshwater ecosystems and suggested that these habitats might offer refuge to lineages that have faced competition and extinction elsewhere [1]. Since the biota of continental slope and abyssal oceanic ecosystems (> 200 m depth) was first documented, these complex habitats have been regarded as oases for very old lineages that have since become depauperate or wholly extinct in shallow-water environments. Classic examples of ancient deep-sea lineages include clades as varied as coelacanths (*Latimeria*) [2], the Vampire Squid *Vampyroteuthis infernalis* [3, 4], crinoids and brittlestars in Echinodermata [5–7], black corals [8], the living graptolite genus *Rhabdopleura* and other colonial hemichordates [9–12], and potentially several lineages of deep-sea isopods [13–15], all of which last share common ancestry with related shallow-water and terrestrial forms well over 200 million years ago. At the same time, recent work has shown that these ancient components of the deep sea biota do not represent a simple accumulation, or sink, of remnant biodiversity over geological time [16]. Rather, many lineages have undergone extensive diversification in continental slope, bathypelagic, and abyssal ecosystems [7, 8, 17, 18].

Vertebrates, which comprise tens of thousands of species distributed across all major ecosystems, underwent their initial diversification in marine habitats during the Ordovician to Devonian Periods, around 480–360 million years ago [19–21]. Despite this ancient history in the ocean, evidence from the fossil record [22] and molecular phylogenetics [23–34] suggests that most living deep marine vertebrate diversity originates from radiations that took place over the last 100 million years. This observation raises the question of whether any living vertebrate diversity in the ocean benthos can truly be considered geologically ancient.

By end of the Devonian, jawed vertebrates had eclipsed the once dominant jawless lineages in diversity and morphological disparity [21, 35, 36]. Presently, only two lineages of jawless vertebrates, lampreys and hagfishes, survive. The resolution of living jawless vertebrates relationships and divergence times is essential for understanding the evolutionary context of living vertebrate diversity [36–42], but these remain disputed [9, 37, 39, 42–55]; only in the past few years have comparative genomic analyses provided strong support for a hagfish-lamprey sister relationship [45, 48].

Hagfishes (Myxiniformes) are a globally-distributed clade of eel-like demersal marine species that are important for comprising a large proportion of vertebrate biomass in ocean floor ecosystems [50, 56]. Although several hagfishes live on the continental shelf [56, 57], most species periodically or permanently occupy continental slope habitats at depths over 200 and up to 3000 m [56–62], where they are ecologically important predators and scavengers [56, 63].

The hagfish body plan, which includes features like a poorly developed eye and skeleton, auxiliary hearts, and a single semicircular canal, departs so much from other vertebrates that this clade has often been placed as the sister to all other living vertebrate clades [35, 42, 49, 53, 64]. Most molecular, morphological, and combined evidence analyses posit that hagfishes are the sister clade of lampreys [9, 37–39, 44–48, 51, 52, 65–68]. However, the interrelationships of hagfishes and the age of living hagfish diversity remain underexplored [37, 38, 40, 43, 69]. This problem is compounded by the poor fossil record of the two living jawless vertebrate clades [36–39, 64–67, 70–74]; only a handful of fossils with well-characterized anatomy are identifiable as early total clade lampreys and hagfishes, and many fossil taxa identified as putative stem-group hagfishes and lampreys [75] may not even be jawless vertebrates [76–78].

Here, we pair data from the fossil record with a dataset including all genera of living hagfishes and ~ 60% of the species diversity to reconstruct the interrelationships of hagfishes and their tempo of diversification. By using fossils to produce a tip-dated phylogeny of living jawless vertebrates, we recover an age for the most recent common ancestor of hagfishes that is over twice as old as previously suggested [37, 38, 51]. Our results show that living hagfishes compose one of the oldest major vertebrate clades, with isolated species potentially representing up to 160 million years of unique evolutionary history. Ancestral state reconstructions support an ancient origin for continental slope ecologies in living hagfishes. These results suggest that hagfishes are an ancient vertebrate clade and clarify their diversification in the deep sea through at least three mass extinctions.

## Methods

### Sequence dataset collection

To produce a comprehensive hypothesis of hagfish interrelationships, we aimed to maximize the number of species sampled. Previous taxonomic and phylogenetic studies concentrated on the mitochondrial genes *COI* and *16S* ribosomal DNA [40, 57, 60, 69, 79] and we gathered sequences of these two genes for all available species (n = 44) on GenBank. Our taxon sampling included two species of *Rubicundus*, two species of *Neomyxine*, 14 species of *Myxine*, and 26 species of *Eptatretus*, with an additional three potentially distinct species of *Eptatretus* from India, Japan, and Korea. Our sample consists of all recognized genera and over 50% of species diversity (https://www.calacademy.org/scientists/projects/eschmeyers-catalog-of-fishes), including the problematic ‘*Notomyxine*’ (= *Myxine*) *tridentiger* and several species previously classified in ‘*Quadratus*’ and ‘*Paramyxine.*’ *Nemamyxine*, which includes two species known from preserved specimens collected in the twentieth century [80], does not have any available genetic material. Thus, its relationships are untestable with molecular phylogenetics. We note that the specimens assigned to *Nemamyxine* have potential affinities to *Rubicundus,* and that the current diagnosis for *Nemamyxine*, which includes an extremely slender body and an anteriorly placed ventral finfold that originates anterior of the ventral gill apertures, is not sufficient to differentiate this lineage from other hagfishes. First, numerous species in *Myxine*, *Rubicundus*, and *Eptatretus*, as well as the extinct species †*Tethymyxine tapirostrum*, show a highly elongated body [37, 40]. Further, slender body depth and high slime pore counts were cited as diagnostic characters for both species of *Nemamyxine*, and both these features are widely distributed among other elongated hagfishes [37, 40]. The placement of the finfold relative to the apertures is a more convincing character. Thus, without genetic material, the phylogenetic position of *Nemamyxine* remains unclear and the available diagnosis is largely uninformative. All sequence data, along with corresponding numbers in GenBank, is included in the Supplementary Data.

### Gene tree inferences

The DNA sequences of the *COI* and *16S* gene were aligned by eye using the translated amino acid sequences as a guide. We also used the online Clustal Omega tool at the EMBL-EBI online resource portal to aid in 16S alignments (www.ebi.ac.uk/Tools/msa/clustalo/). Phylogenies were inferred using both maximum likelihood and Bayesian methods. We used the gnathostomes *Polypterus ornatipinnis*, *Protopterus annectens*, and *Callorhincus milii* and the lampreys *Geotria australis*, *Petromyzon marinus*, and *Lampetra fluviatilis* as outgroups for the analysis of the *COI* gene and rooted the *16 s* tree on *Rubicundus*. Maximum likelihood analyses were conducted on each of the COI and 16S alignments and with both concatenated together using the software IQTREE v. 2.2.0.3 [81] with branch support assessed using 100 standard bootstrap replicates. We allowed IQTREE to find the optimal partitions and molecular evolutionary models using AIC values via ModelFinder [82]. Preferred models were the TN93 + F + I + G4 model for *COI* and TIM2 + F + I + G4 for *16S*. Bayesian analyses were conducted using the program MrBayes v. 3.2 [83] using the GTR + G evolutionary model. Analyses were run for 1.0 X 10^7^ generations, and two simultaneous runs were conducted within each of four chains. We assessed chain convergence and stationarity by inspecting chain likelihoods and monitoring average standard deviations of split frequencies between the two runs to ensure values less than 5.0 × 10^–3^ after 1.0 X 10^6^ generations. We discarded the first 50% of sampled generations as burn-in and summarized the posterior tree set in a 50% majority-rule tree.

### Bayesian tip-dating analyses

We jointly estimated the phylogenetic relationships and divergence times of hagfishes and a subset of lampreys using a tip-dating approach as implemented in the program BEAST 2.6.6 [84, 85] using the fossilized birth–death (FBD) model [86]. Lampreys were included to allow for use of several fossils as tip-calibrations and because they are phylogenetically proximal to hagfishes among living vertebrates. We generated modified input molecular data files that included all hagfish species for which DNA sequences were available and our included fossil tip calibrators whose phylogenetic positions we constrained based on the results of previous studies using monophyletic MRCA priors. A complete list of calibrations, along with age and placement justifications, is included in the Supplementary Information. In total, we selected two fossil lampreys (one total group and one crown group), one crown-group fossil hagfish, and putative stem-hagfish †*Myxinikela siroka* as fossil tip calibrators. We set the origin prior at 439.0 Ma (bounds of 400 and 600 Ma), which is the age of the oldest gnathostome †*Fanjingshania renovata* from the early Silurian of China [87], to account for the uncertainty surrounding the monophyly of cyclostomes. We applied a TN93 model of nucleotide evolution to our *COI* sequence data and a GTR model to our *16S* sequence data following the model choices of the IQTREE analysis. We set the rho parameter of the FBD model to 0.57, which is the proportion of known living species included in the dataset, and set the diversification rate prior to 0.1 based on the ratio of living species in the dataset to the origin prior, with bounds of 0.0 and infinity. We used a gamma prior with a default value of 1.0 for the mean and 0.33 for the standard deviation, and relaxed uncorrelated lognormal clock model. Two BEAST runs were conducted over 1.0 X 10^8^ generations with 1.0 X 10^7^ pre-burnin, and convergence of the posteriors was checked using Tracer v. 1.7.1 [88]. We combined the posterior tree sets in LogCombiner v. 2.6.6 with 10% burnins and summarized them in a maximum clade credibility (MCC) tree with median node heights using TreeAnnotator 2.6.4. We conducted a set of three BEAST analyses without using the putative stem-hagfish †*Myxinikela siroka* as a tip calibration for the hagfish total clade to test its influence on divergence time estimates. Finally, we conducted BEAST analyses of including and excluding †*Myxinikela siroka* as a tip calibration but only sampling from prior values, in order to test whether our divergence time estimates for the analyses using sequence data were driven by only the priors. We compared the posterior mean and 95% highest posterior density intervals for the divergence times of selected major hagfish clades across both iterations of our BEAST analyses (Fig. 2). All input xml files and output files for both BEAST run iterations are included in the Supplementary Information.

In order to assess the phylogenetic informativeness of the sequences we used for reconstructing ancient relationships in hagfishes, we used the program hyphy as implemented through the phydesign online tool [89, 90] to plot the phylogenetic informativeness of both mitochondrial loci used in this study for reconstructing hagfish relationships through time.

### Age comparisons

We compared the estimated ages of hagfish divergences to previous ones [37, 51] (Table S1) and the ages of the hagfish and lamprey crown groups estimated from the Bayesian tip-dating analysis including †*Myxinikela siroka* to other major vertebrate clades by extracting crown age estimates from TimeTree.org [91] We recorded the age of the MRCA of living gars from our recent relaxed molecular clock estimation [92]. The complete list of crown ages are listed in Table S2.

### Ancestral habitat reconstruction

**Fig. 1:**
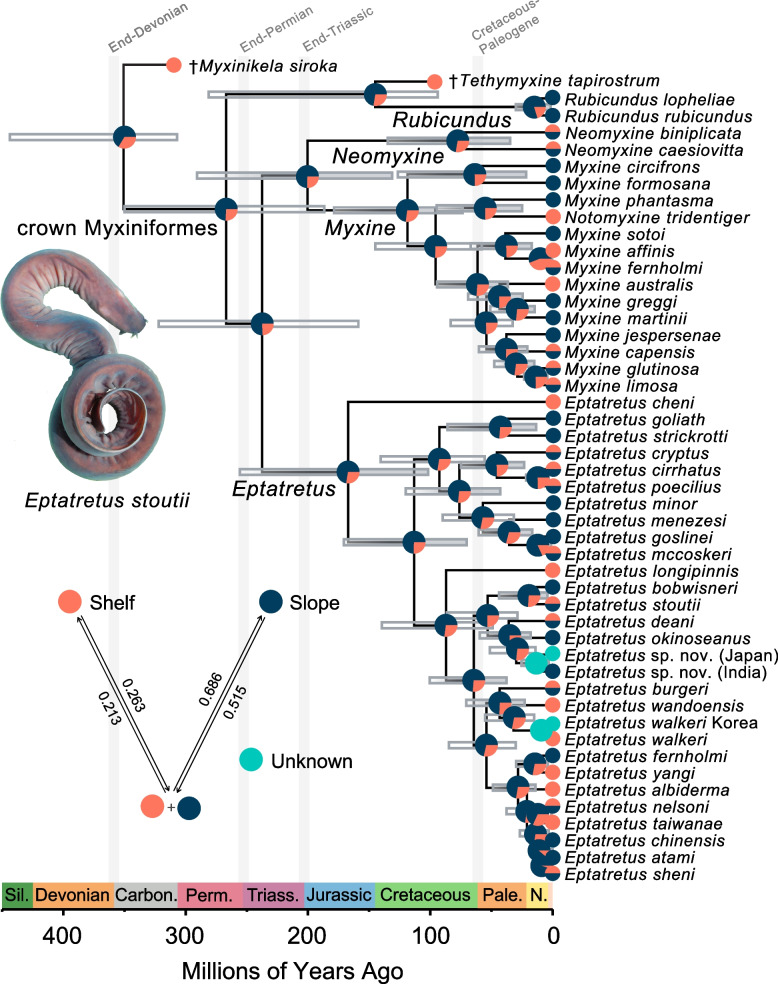
Hagfish phylogeny and tempo of diversification. Tip-dated Bayesian maximum clade credibility phylogeny of jawless fishes from two independent runs in BEAST 2.6.6 showing the interrelationships of the major lineages of hagfishes. Bars indicate 95% highest posterior density (HPD) intervals for divergence times at nodes. Outgroups not shown. Grey bars are at nodes supported by posterior values of 0.90 or more, clear bars are at nodes supported by posterior values of 0.89 or less. Gray columns indicate mass extinction events. Dagger (†) indicates extinct species known from the fossil record. Pie charts indicate ancestral state reconstructions of habitat for each node, where purple represents the probability of a slope component (either slope or shelf-slope) at each node and salmon indicates the probability of continental shelf habitat being ancestral. Inset includes the transition matrix from the polymorphic character ancestral reconstruction analysis (note that purple here is exclusively slope, as opposed to purple denoting slope/shelf-slope at nodes in the phylogeny). Photograph of *Eptatretus stoutii* is courtesy Douglas Fudge

In order to assess the evolution of depth preference among hagfishes, we assembled a dataset from FishBase and the literature (Table S3) on observed depth ranges for hagfish species. Values of 200 m or less were coded as continental shelf ranges, and values of over 200 m were coded as continental slope regions following the literature [8, 93, 94]. Most studies attempting to reconstruct habitat preference evolution have either divided habitat characters into a discrete states [7, 93, 95–98] or treated different habitats as independent areas [8]. However, because ecological character states can form a spectrum and species can show polymorphic ecological states (i.e., appearing in more than one habitat), modeling overlap as discrete characters (e.g., denoting slope, shelf, and shelf-slope as distinct, discrete characters) is nonideal [99]. To account for this problem, we used the R package phytools [100] to perform stochastic ancestral character mapping where habitat preference was treated as a polymorphic character using the fitpolyMk function with unordered transition rates (“ARD” model) and the root prior distribution π proposed by Fitzjohn et al. [101]. Given that there were only two primary states (shelf, slope), we selected the ARD model a priori because it is the most generalized unordered model available for polymorphic traits in phytools. The results of fitpolyMk were used for stochastic character mapping over 1000 simulated topologies, and the results were summarized into a single reconstruction (Fig. 1). All code necessary to replicate this analysis is in the supplement.

## Results

### Phylogeny and divergence times of Myxiniformes

Maximum likelihood and Bayesian analyses of our hagfish sequence dataset supported the reciprocal monophyly of three major lineages (Fig. 1, Figure S1-S4): Rubicundinae, Eptatretinae and Myxininae. *Neomyxine* is resolved as the sister lineage of *Myxine* (Fig. 1a, Figure S1, Figure S2, Figure S3). The resolution of Rubicundinae as the sister clade of all other hagfishes is consistent with some previous phylogenetic analyses [37, 38, 40, 60] but incongruent with other studies that variably resolve *Neomyxine* as the sister lineage of all other hagfishes [79]. Our phylogenetic results affirm the synonymy of several genera of hagfishes, including *Quadratus* and *Paramyxine*, with *Eptatretus* and the enigmatic *Notomyxine tridentiger* with *Myxine* (Fig. 1a) [40, 57, 79].

**Fig. 2:**
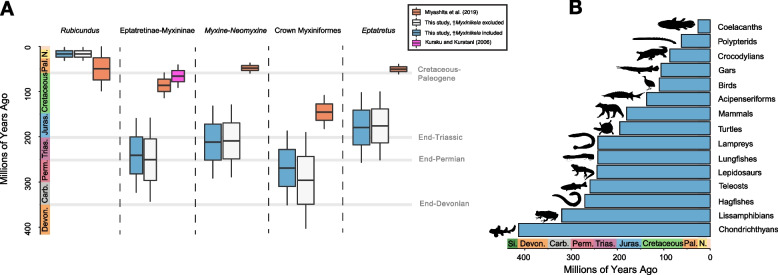
The Paleozoic age of the hagfish radiation. Boxplots (**A**) generated using the R package ggplot2 for divergence times of major hagfish lineages under different fossil calibration schemes and those reported in a previous study. Note the similar ages estimated for these lineages regardless of the inclusion of †*Myxinikela siroka* as a fossil tip calibration, and the much older ages for all major hagfish clades except Rubicundinae estimated in this study compared to previous estimates based on smaller molecular datasets and less-inclusive taxon sampling. Panel (**B**) shows the ages of major vertebrate crown groups. Hagfishes are the third oldest, exceeded only by the far more species rich and morphologically disparate lissamphibians (frogs, salamanders, and caecilians) and chondrichthyans (sharks, rays, skates, and chimaeras). Silhouettes are public from phylopic.org, Wikimedia commons, and by CDB

†*Gilpichthys greenei*, known from numerous poorly preserved specimens from the Mazon Creek, from all of our analyses. Recent phylogenetic analyses place †*G. greenei* as an indeterminate jawless vertebrate [37, 49, 66, 67] or as the most stemward hagfish [38, 53, 73]. Because †*G. greenei* is classically united with Myxiniformes based on the *absence* of features like mineralized teeth, the placement of this taxon among early vertebrates might be biased by the phenomenon of stemward slippage, whereby decomposition of the body can lead to erroneous interpretations of a fossil’s phylogenetic affinities [102, 103].

Our tip-dated Bayesian analyses of hagfish phylogeny consistently recovered Paleozoic ages for the major hagfish crown clades (Fig. 1; Fig. 2). Relaxed clock analyses using BEAST with the inclusion and exclusion of †*Myxinikela siroka* resulted in closely comparable posterior divergence time estimates for all major hagfish lineages (Fig. 2A), demonstrating that the tip-dating scheme employed in this study is robust to the use of putative Mazon Creek hagfishes as calibrations. This is especially important given that we excluded the wildcard taxon.

**Fig. 3:**
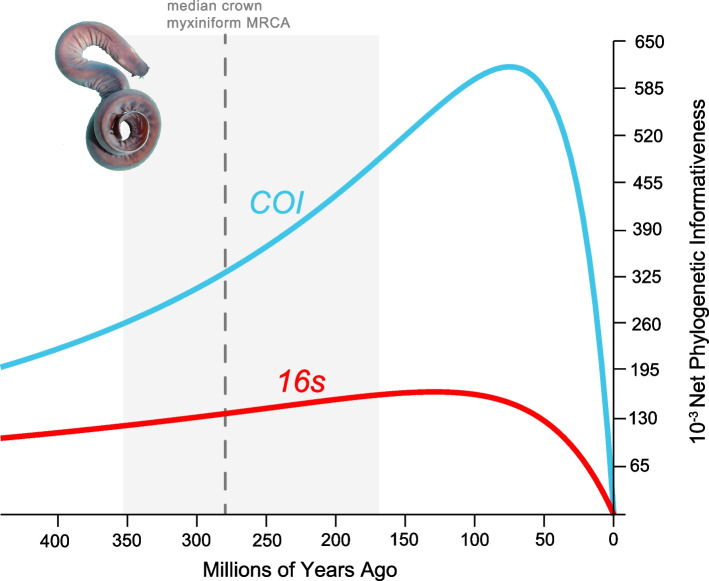
Phylogenetic Informativeness Analysis. Plot shows the phylogenetic informativeness of both mitochondrial loci used in this study through time. The grey shaded area represents the 95% HPD interval for crown Myxiniformes found in the tip-dated phylogeny where †*Myxinikela siroka* was used as a fossil tip calibration. Photograph of *Eptatretus stoutii* is courtesy Douglas Fudge

Across different analyses, we consistently estimate an Early Permian origin for the hagfish crown and an early Triassic age for the split between *Eptatretus* and Myxininae (Fig. 1, Fig. 2A). These age ranges are substantially older than previous estimates of hagfish diversification (Fig. 2A), which invariably place the origins of the major living hagfish clades far later in the middle to Late Cretaceous [10, 17, 33]. Although we use mitochondrial gene sequences (mtDNA), which have been shown to overestimate divergence times for ray-finned fish ingroups relative to nuclear DNA sequences [104], sequence data type cannot explain the discrepancy between our results and previous estimates, which also used mtDNA [37]. Instead, our results are best explained by a combination of more extensive hagfish species sampling (including several deeply-divergent singletons like *Eptatretus cheni*) and more stringent criteria for including fossils as tip calibrations. For example, †*Gilpichthys greenei*, a problematic fossil chordate [37, 38, 73] from the Mazon Creek Lagerstätten, was excluded in our analyses but placed as a stem-lamprey with little evidence in the previous phylogenetic analyses [37]. Phylogenetic informativeness analyses [89] suggest that, while the informativeness of *COI* declines for hagfish divergences over 100 million years ago, *16S* provides steady (albeit reduced relative to *COI*) phylogenetic information across the age of hagfish evolutionary history (Fig. 3).

The revised timescale of hagfish diversification (Figs. 1 and 2) reduces the long branch separating living hagfishes from other vertebrates by over 120 million years [9, 10, 17, 19, 33, 44] and demonstrates that the hagfish crown clade has persisted through the last three mass extinctions. Further, our age estimates place crown hagfishes among the oldest vertebrate crown clades (Fig. 2B), suggesting that living hagfish diversity is far more ancient than most other marine vertebrates. These age estimates are not attributable to our choice of prior settings, as runs sampling only from priors produce far older ages for hagfish divergence times.

### Ancestral habitats of hagfishes

Ancestral habitat reconstruction along the time-calibrated phylogeny indicates that hagfishes have occupied continental slope habitats with depths of over 200 m since the origins of the crown group during the late Paleozoic (Fig. 1). Although there is a high degree of variability in living hagfish habitat utilization and fossil hagfishes are only known from estuarine and continental shelf settings (depth < 200 m) [37, 38], polymorphic character ancestral state reconstruction favors a continental slope component (either slope or shelf + slope) as ancestral for nearly all deep nodes in crown hagfish phylogeny (Fig. 1; note the root node for Myxiniforms is less clearly resolved). Continental slope components are more strongly inferred for the ancestral habitat of crown *Rubicundus* and several recent divergences inside *Eptatretus* (Fig. 1), whereas the continental shelf appears to be supported as the ancestral habitat for the clade containing *Myxine fernholmi* and *Myxine affinis*.

## Discussion

### Paleozoic origins of hagfish diversity

Since the end of the Triassic, only hagfishes and lampreys have persisted as the survivors of the once more diverse grade of jawless vertebrates [36, 37, 53–55]. This accident of deep time makes these two clades particularly important for comparative studies of phylogenetic relationships of early vertebrates and the context of their diversification [36, 37, 48, 51]. Because the anatomy of lampreys and hagfishes diverges considerably from other living vertebrates and both lineages have poor fossil records, molecular phylogenetics provides key information for reconstructing the relationships and timescale of diversification in living jawless vertebrates.

Our hypothesis of hagfish phylogeny and relaxed molecular clock divergence time estimates reveals an ancient origin of crown Myxiniformes during the Permian period (Fig. 1, Fig. 2). We infer that the initial divergences of living hagfishes occurred in the Paleozoic and earliest Mesozoic, only 20 to 30 million years after the first putative hagfishes appear in the fossil record [38]. This old age for hagfish diversity highlights a hidden period of jawless fish radiation that followed the extinction of ‘ostracoderms,’ a grade of armored, jawless fishes that formed the dominant assemblage of vertebrates until the Devonian [35, 36, 53, 54, 105].

The body plan of hagfishes remains highly conserved and includes specializations such as deskeletonization [37, 106], a rudimentary visual system [107, 108], burrowing and knotting feeding behavior [56, 63], and tolerance to high ammonia concentrations, such as those from carrion [109]. The low rate of morphological change observed in hagfishes provides a notable contrast to similarly old clades of vertebrates that exhibit high phenotypic diversity and species richness, such as teleosts, chondrichthyans, and lissamphibians (Fig. 3a-c) [24, 25, 110–115]. The low morphological disparity observed among living hagfishes and the ancient age inferred for the crown also imply that the specialized anatomy of this clade appeared by the end of the Paleozoic.

The tempo of hagfish diversification contrasts with the pattern observed in lampreys, the only other living clade of jawless vertebrates [36, 39], which include numerous regional radiations that have diversified over the past 100 million years [36]. In contrast, our time-calibrated phylogeny of hagfishes infers an average evolutionary interval of 31.6 million years of common ancestry for individual hagfish species, which is considerably higher than the corresponding values for cartilaginous and bony fishes [115] or the one-to-two-million-year divergences of most lamprey species pairs [36]. The most isolated single branch on the hagfish tree leads to *Eptatretus cheni* (Fig. 1a), which we estimate diverged from all other species of *Eptatretus* during the Jurassic Period (Fig. 1: median MRCA age = 167.24 Ma, 95% HPD: 101.77, 255.65 Ma). The age of this single branch is comparable to the most evolutionarily isolated species among sharks, rays, and chimeras [115], as well as long branches like the Tuatara *Sphenodon punctatus* [111, 116] and the Salamanderfish *Lepidogalaxias salamandroides* [26, 28, 30].

### Hagfishes are ancient inhabitants of continental slope settings

The phylogenetic hypothesis of hagfishes presented in this paper highlights them as a trove of ancient vertebrate evolutionary history hidden in oceanic demersal habitats. The true diversity of living hagfishes remains a frontier of biological research but is challenged by their deep marine habitats. For example, *Rubicundus* is the living sister lineage of all other hagfishes (Fig. 1, Figure S1-S3) and has a nearly cosmopolitan geographic distribution, but was identified as a distinct genus and described in the last decade [40]. In addition, species discovery in hagfishes continues at a pace as 15% of recognized species in the clade were described over the past ten years [117]; several new forms were recovered at depths in the thousands of meters [60].

Ancestral state reconstructions of habitat along the time-calibrated hagfish phylogeny that we present in this paper (Fig. 1) demonstrate an ancient history of continental slope habitat use in Myxiniformes. Despite levels of uncertainty introduced by the variability of habitat use among hagfish subclades (including among species; Fig. 1; [62]), we infer that hagfishes have accessed continental slope settings in the deep sea since at least the Permian period (Fig. 1), with several subclades in *Myxine* and *Eptatretus* diversifying recently in continental shelf settings. This makes hagfishes the vertebrate clade with the most ancient history in the deep sea, far outpacing the ages of deep-sea diversifications in teleost fishes [23, 24, 26, 31, 32, 34, 93] or chondrichthyans [118]. The deep-sea habitats reconstructed as ancestral for hagfishes complicate the current picture of early vertebrate diversification. Although evidence from the fossil record, including fossil hagfishes [38], indicate that early vertebrates primarily diversified in coastal environments [21, 119, 120], our results suggest that exploration of the deep sea was occurring in at least one clade during the Paleozoic.

### Deep sea cradles and refugia of hagfish diversity

One possibility is that demersal marine habitats have served as a refugium for the diversity of hagfishes. Although similarly old marine vertebrate clades such as cartilaginous fishes did experience declines in phenotypic disparity, they show less pronounced species turnover than other lineages during major events like the Cretaceous-Paleogene mass extinction [121–124]. Some of the oldest surviving chondrichthyan clades, such as the goblin shark, frilled and sevengill sharks, and the chimeras and ratfishes, occupy the deep sea, underscoring these habitats as a refugium of ancient diversity in cartilaginous fishes [115]. Consequently, the evolutionary history of hagfishes provides an intriguing parallel with long-lived, species-poor lineages from freshwater ecosystems often referred to as living fossils, which are classically thought to have been buffered from extinction by their environments [1, 92, 125–128].

At the same time, our time-calibrated phylogeny suggests that considerable speciation (Fig. 1) has taken place in continental slope environments over the past 100 million years. Despite the old age of the hagfish crown group and the high proportion of species with ancient unique evolutionary histories, our results highlight that Myxiniformes includes ancient species diversity that has also undergone significant diversification since first appearing in the marine benthos.

## Conclusions

Vertebrate diversity in the deep sea is mostly young (< 100 million years old) relative to the ancient ages of many continental slope and abyssal invertebrate radiations. This observation contrasts with the hypothesis that the deep sea has been a refugium for animal diversity, and also begs the question of whether any deep sea vertebrates are ancient endemics. Here, by reconstructing the phylogeny and divergence times of hagfishes, we substantiate this clade as a truly ancient lineage with a long history in continental slope settings (> 200 m depth). The evolutionary history of hagfishes reconstructed in this paper markedly contrasts with that of the only other surviving jawless vertebrate clade, the lampreys, and implies that vertebrate colonization of habitats classically considered part of the deep sea [94] has taken place since the Paleozoic. Our results highlight hagfishes as a unique vector of ancient vertebrate diversity that has persisted and diversified in the deep sea.

### Supplementary Information

Supplementary Material 1.

## Data Availability

All data is in the supplementary information submitted with this article. No new sequence data were generated for this study.
