# Correction: Colonization of the ocean floor by jawless vertebrates across three mass extinctions

**DOI:** 10.1186/s12862-024-02286-3

**Published:** 2024-07-12

**Authors:** Chase Doran Brownstein, Thomas J Near

**Affiliations:** 1 Department of Ecology and Evolutionary Biology, Yale University, New Haven, CT 06511 USA; 2 Yale Peabody Museum, Yale University, New Haven, CT 06511 USA

**Correction to: BMC Ecology and Evolution (2024) 24:79**

10.1186/s12862-024-02253-y

After publication of this article [1], it was brought to our attention that there are two minor errors:

The second author’s name Thomas Near should be Thomas J Near.

The last sentence of the Result section in the Abstract should be removed: Yet, we show that hagfishes have experienced marked body size diversification over the last hundred million years, contrasting with a view of this clade as morphologically stagnant.

The original publication has been corrected.
