# Supplementary material for: Colonization of the ocean floor by jawless vertebrates across three mass extinctions
Source: BMC Ecol Evol. 2024 Jun 13;24:79. doi: 10.1186/s12862-024-02253-y (PMC11170801; doi:10.1186/s12862-024-02253-y)
Supplement: Supplementary file 1 — Supplementary Material 1. [file 12862_2024_2253_MOESM1_ESM.zip › Figure_S5.pdf]

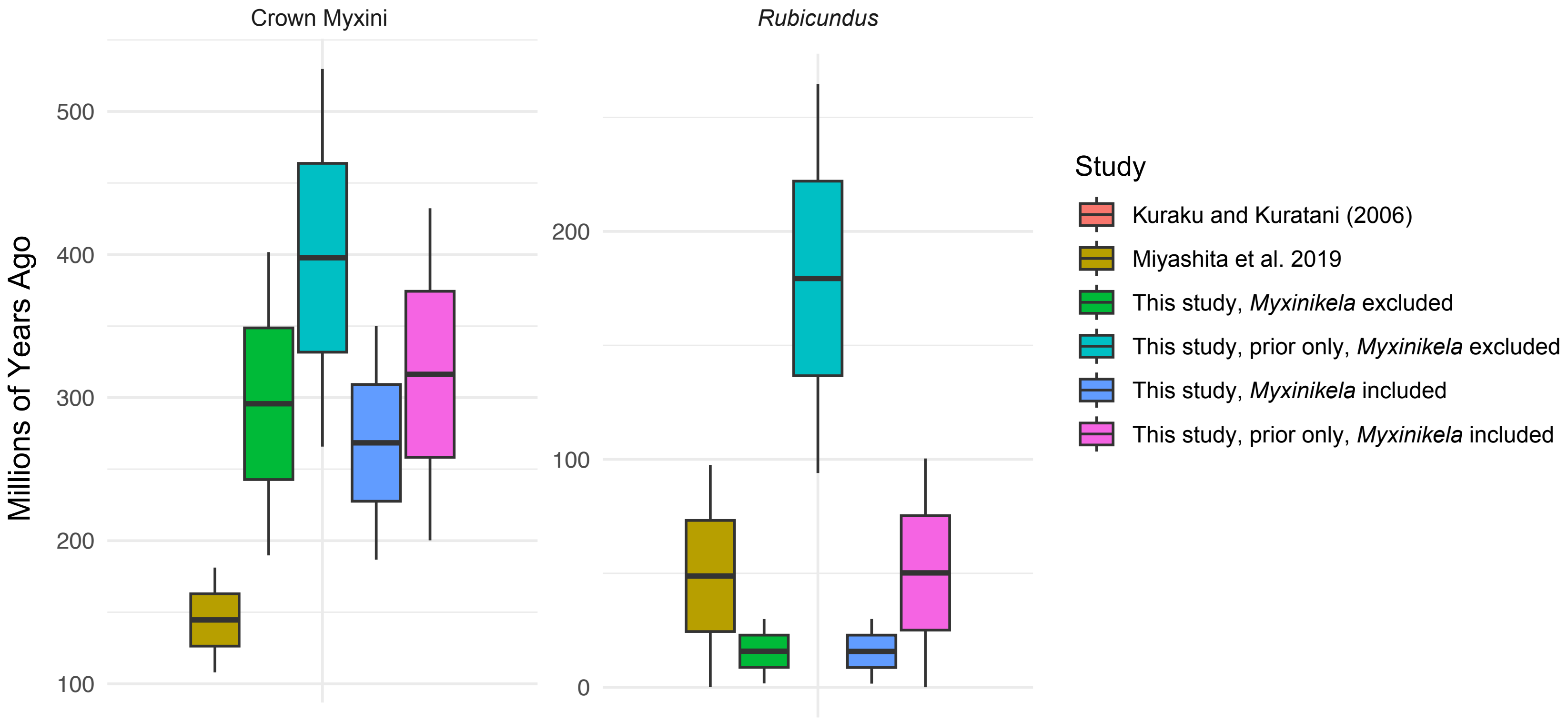

Crown Myxini
Rubicundus
Millions of Years Ago
500
400
300
200
100
200
100
0
Study
Kuraku and Kuratani (2006)
Miyashita et al. 2019
This study, Myxinikela excluded
This study, prior only, Myxinikela excluded
This study, Myxinikela included
This study, prior only, Myxinikela included
