# Supplementary material for: Colonization of the ocean floor by jawless vertebrates across three mass extinctions
Source: BMC Ecol Evol. 2024 Jun 13;24:79. doi: 10.1186/s12862-024-02253-y (PMC11170801; doi:10.1186/s12862-024-02253-y)
Supplement: Supplementary file 1 — Supplementary Material 1. [file 12862_2024_2253_MOESM1_ESM.zip › Supplementary_text.docx]

**Supplementary Text.**

**Fossil Calibration Justifications.**

**†*Priscomyzon rinensis***

**Resolution in Phylogenetic Analyses**: †*Priscomyzon rinensis* is invariably supported as a stem-group lamprey in all phylogenetic analyses including this taxon, regardless of analysis type (Bayesian, parsimony, time-calibrated Bayesian) or taxon sample [1–5].

**Stratigraphy**: Witpoort Formation, Famennian of Waterloo Farm, Grahamstown, South Africa [5]. The lower bound is therefore 359.3 Ma [6]. This age is used in previous studies [2–4].

**Fossil tip age**: 359.3 Ma.

**†*Mesomyzon mengae***

**Resolution in Phylogenetic Analyses**: †*Mesomyzon mengae* is supported as a crown-group lamprey sister to Petromyzontidae in parsimony and Bayesian analyses of morphological data and combined morphological and molecular data [2]. It is supported as a lamprey near the crown group or within an unresolved polytomy in the crown in various analyses [1,4].

**Stratigraphy**: Yixian Formation, Barremian-Aptian of Liaoning Province, China [7]. This is approximately 125.0 Ma [8].

**Fossil tip age**: 125.0 Ma.

**†*Tethymyxine tapirostrum***

**Resolution in Phylogenetic Analyses:** †*Tethymyxine tapirostrum* is invariably supported as a pan-Rubicundine hagfish in the crown group in all phylogenetic analyses including this taxon, regardless of analysis type (Bayesian, parsimony, time-calibrated Bayesian) or taxon sample [3,4]. Differential coding of this taxon does result in it nesting in a polytomy with other crown-group hagfishes [4]. However, several features, including a tapering rostrum leading up to the elongated nasohypophyseal aperture and an elongated body with numerous slime glands, strongly unite it with *Rubicundus* spp. [4].

**Stratigraphy**: Hâdjula Lagerstätte, Cenomanian, 10 km east of Byblos, Lebanon. The horizon dates to approximately 94.0 Ma [8].

**Fossil tip age:** 94.0 Ma.

**†*Myxinikela siroka***

**Resolution in Phylogenetic Analyses:** †*Myxinikela siroka* is invariably supported as a stem hagfish in all phylogenetic analyses including this taxon, regardless of analysis type (Bayesian, parsimony, time-calibrated Bayesian) or taxon sample [3,4,9,10]. A recent reevaluation of this taxon [3], despite agreeing with this consensus, cast doubt on the presence of several putative hagfish synapomorphies that Bardack [11,12] identified in †*M. siroka*. These include the putative nasohypophyseal barbels and aperture, which are labeled as “?” in figures in Miyashita [3] despite being coded in the phylogenetic analysis of that study. The posteriorly-placed branchial basket and dorsal migration of the eyes of †*M. siroka* could also be taphonomic artifacts owing to the displacement of the eyes postmortem and disintegration of the anterior branchial basket; Miyashita [3] identifies individual arches along the basket, but the mangled state of this feature ought to preclude such precision in anatomical character description of the †*M. siroka* holotype. Indeed, Bardack [11,12] identified these structures as blood vessels. The presence of a gallbladder, an organ absent in living lampreys, in †*M. siroka* [3] is also questionable, as Bardack [11,12] identified the same structure as the liver. Similarly, a cardinal heart is identified in †*M. siroka* without much justification [3], such as the identification of iron-rich sediment indicative of heme groups and thus concentrated blood remnants. Interestingly, †*Myxinikela siroka* shows numerous features, including the presence of a branchial basket and developed, pigmented eyes, a differentiated midline finfold, and a distinct esophageal tract, with lampreys; these were considered transitional features in previous studies. †*M. siroka* occurs in a near-shore estuarine environment, whereas all other extinct and living hagfishes are marine animals. To account for the possibility that †*M. siroka* is not a stem-hagfish, we ran two BEAST analyses excluding this taxon as a tip calibration (see the Methods Section).

**Stratigraphy**: Francis Creek Shale, Moscovian of Will-Kankakee counties, Illinois, USA [3].

**Fossil tip age:** 307.0 Ma.

**Supplementary Figure and Table Captions.**

**Figure S1. Gene trees for the *COI* dataset.** Trees from (A) maximum likelihood inference in IQTREE and (B) Bayesian estimation in MrBayes of hagfish phylogeny using the *COI* dataset.

Support values under 80 (A) or 0.8 (B) not shown.

**Figure S2. Gene trees for the *16s* dataset.** Trees from (A) maximum likelihood inference in IQTREE and (B) Bayesian estimation in MrBayes of hagfish phylogeny using the *16s* dataset. Support values under 80 (A) or 0.8 (B) not shown.

**Figure S3. Concatenated gene tree.** Tree from (A) maximum likelihood inference in IQTREE of hagfish phylogeny using the concatenated dataset. Support values under 80 (A) or 0.8 (B) not shown.

**Figure S4. Testing †*Myxinikela siroka* as a hagfish total group tip calibration.** Tip-dated Bayesian maximum clade credibility trees of jawless fishes from three independent runs in BEAST 2.6.6 without †*Myxinikela siroka* included as a stem hagfish tip calibration, showing the interrelationships of the major hagfish clades. Bars at nodes indicate 95% highest posterior density (HPD) intervals for divergence times at nodes. Grey bars are at nodes supported by posterior values of 0.90 or more, clear bars are at nodes supported by posterior values of 0.89 or less. Dagger (†) indicates extinct species.

**Figure S5. Testing the influence of prior calibrations.** Figure shows boxplots of the divergence times of key hagfish nodes comparable across different BEAST analyses in this study, as well as previous ones. The divergence time estimates found using only priors are markedly older than those estimated using priors and DNA sequences.

**Table S1.** Hagfish body length data.

**Table S2.** Hagfish crown clade and subclade age estimates from different analyses and studies.

**Table S3.** Vertebrate crown clade age estimates from different studies.

**Table S4.** Input hagfish habitat data.

**References.**

1. Wu F, Janvier P, Zhang C. 2023 The rise of predation in Jurassic lampreys. *Nat Commun* **14**, 6652. (doi:10.1038/s41467-023-42251-0)

2. Brownstein CD, Near TJ. 2023 Phylogenetics and the Cenozoic radiation of lampreys. *Current Biology* **33**, 397-404.e3. (doi:10.1016/j.cub.2022.12.018)

3. Miyashita T. 2020 A Paleozoic stem hagfish Myxinikela siroka — revised anatomy and implications for evolution of the living jawless vertebrate lineages. *Can. J. Zool.* **98**, 850–865. (doi:10.1139/cjz-2020-0046)

4. Miyashita T *et al.* 2019 Hagfish from the Cretaceous Tethys Sea and a reconciliation of the morphological–molecular conflict in early vertebrate phylogeny. *Proceedings of the National Academy of Sciences* **116**, 2146–2151. (doi:10.1073/pnas.1814794116)

5. Gess RW, Coates MI, Rubidge BS. 2006 A lamprey from the Devonian period of South Africa. *Nature* **443**, 981–984. (doi:10.1038/nature05150)

6. Ogg JG, Ogg GM, Gradstein FM. 2016 8 - Devonian. In *A Concise Geologic Time Scale* (eds JG Ogg, GM Ogg, FM Gradstein), pp. 85–98. Elsevier. (doi:10.1016/B978-0-444-59467-9.00008-X)

7. Wu F, Chang M-M, Janvier P. 2021 A new look at the Cretaceous Lamprey Mesomyzon Chang, Zhang & Miao, 2006 from the Jehol Biota. *geod* **43**, 1293–1307. (doi:10.5252/geodiversitas2021v43a23)

8. Ogg JG, Ogg GM, Gradstein FM. 2016 13 - Cretaceous. In *A Concise Geologic Time Scale* (eds JG Ogg, GM Ogg, FM Gradstein), pp. 167–186. Elsevier. (doi:10.1016/B978-0-444-59467-9.00013-3)

9. McCoy VE *et al.* 2016 The ‘Tully monster’ is a vertebrate. *Nature* **532**, 496–499. (doi:10.1038/nature16992)

10. Dearden RP, Lanzetti A, Giles S, Johanson Z, Jones AS, Lautenschlager S, Randle E, Sansom IJ. 2023 The oldest three-dimensionally preserved vertebrate neurocranium. *Nature* **621**, 782–787. (doi:10.1038/s41586-023-06538-y)

11. Bardack D. 1991 First Fossil Hagfish (Myxinoidea): A Record from the Pennsylvanian of Illinois. *Science* **254**, 701–703. (doi:10.1126/science.254.5032.701)

12. Bardack D. 1998 Relationships of Living and Fossil Hagfishes. In *The Biology of Hagfishes* (eds JM Jørgensen, JP Lomholt, RE Weber, H Malte), pp. 3–14. Dordrecht: Springer Netherlands. (doi:10.1007/978-94-011-5834-3_1)
