# Supplementary figures and images for: Colonization of the ocean floor by jawless vertebrates across three mass extinctions
Source: BMC Ecol Evol. 2024 Jun 13;24:79. doi: 10.1186/s12862-024-02253-y (PMC11170801; doi:10.1186/s12862-024-02253-y)

**A**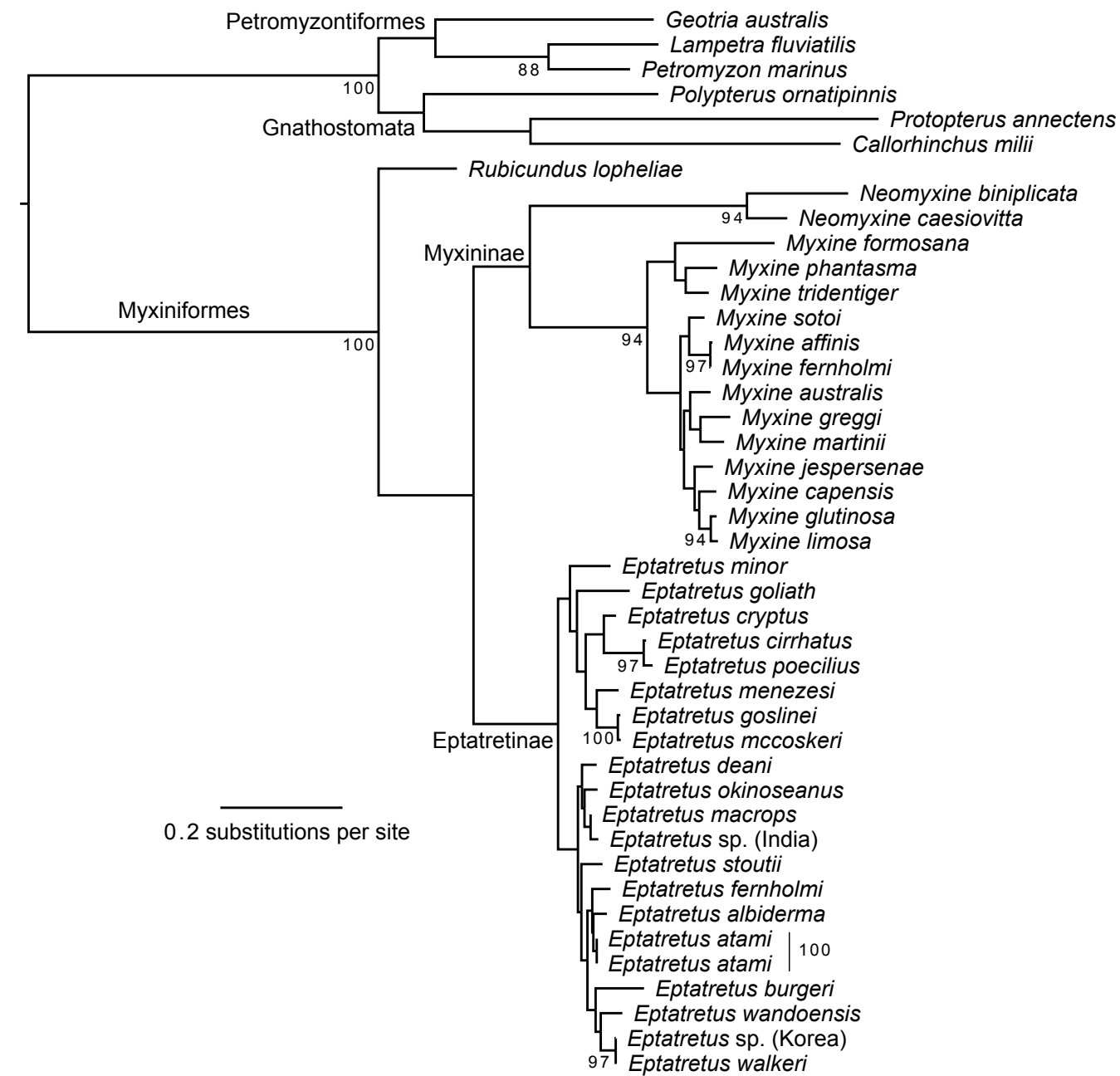**B**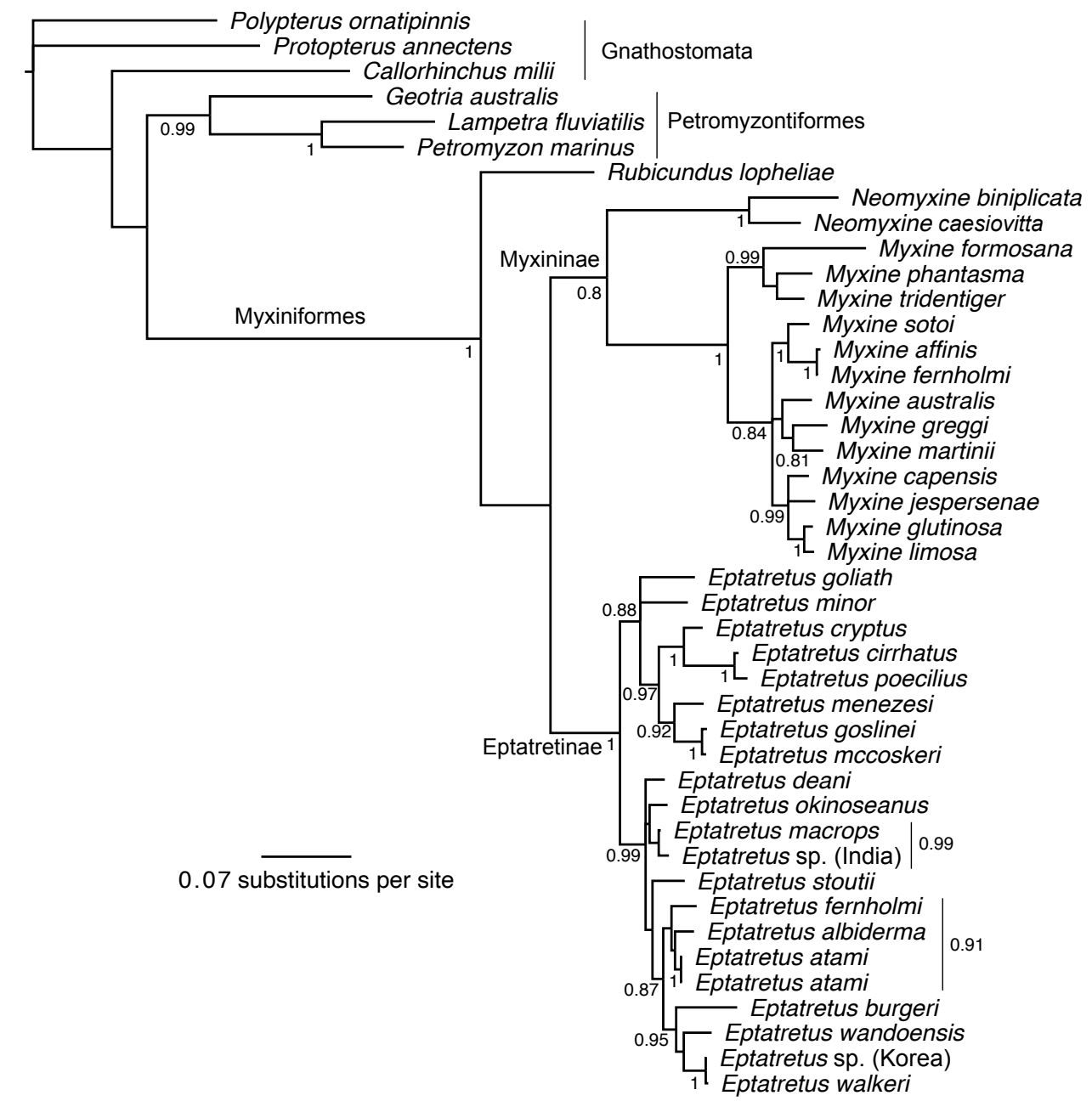

Supplement: Supplementary file 1 — Supplementary Material 1. [file 12862_2024_2253_MOESM1_ESM.zip › Figure S1.pdf]

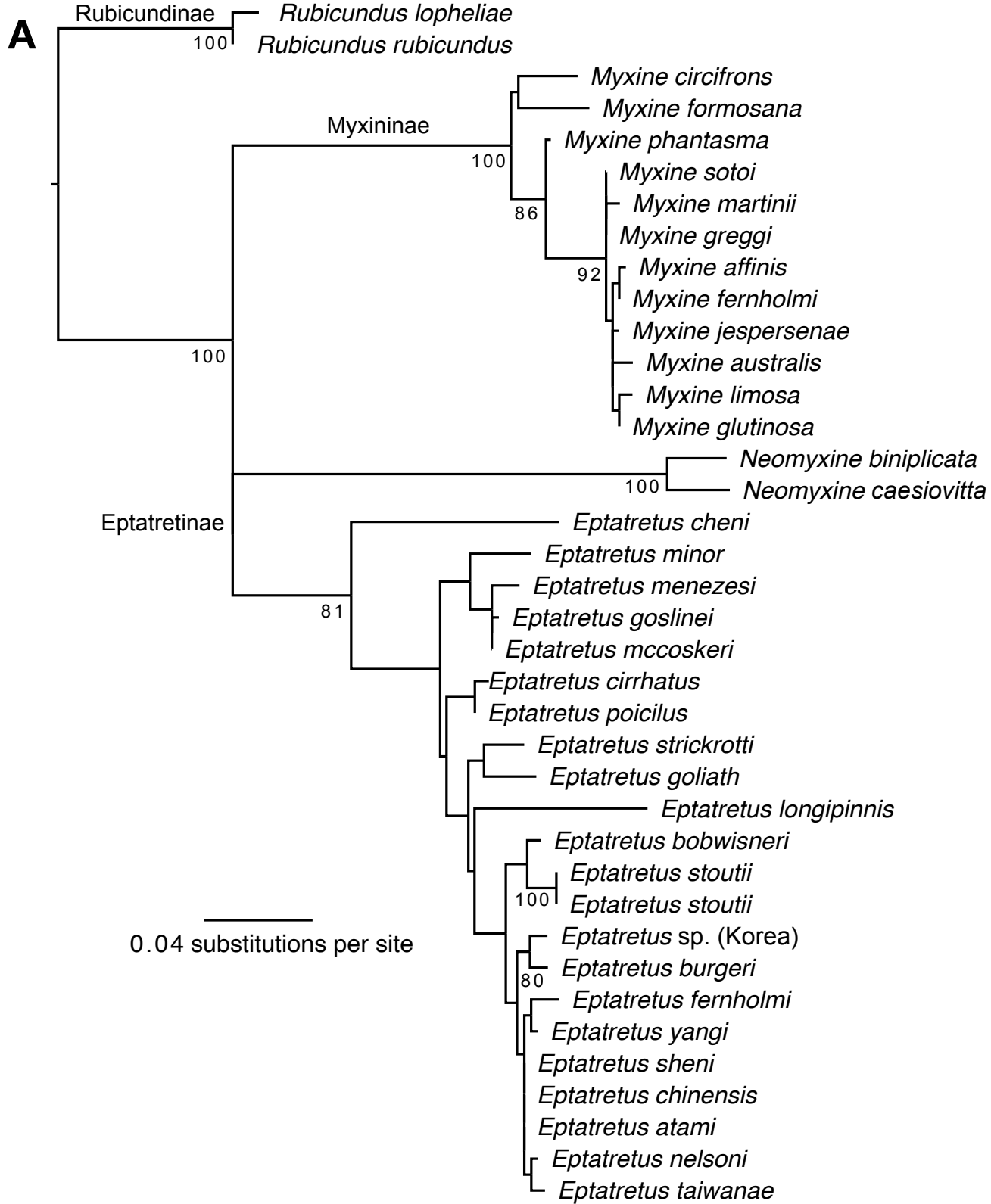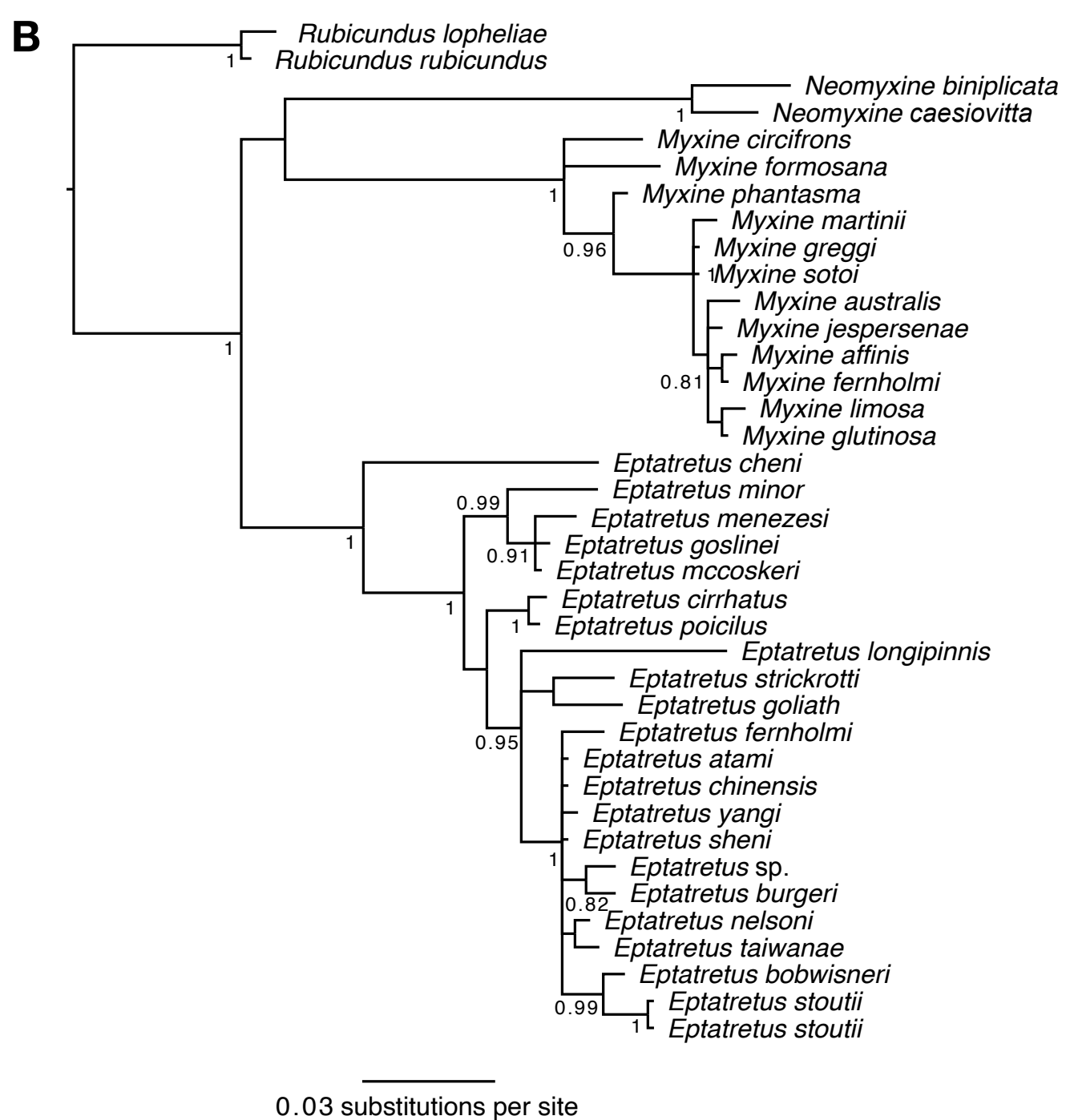

Supplement: Supplementary file 1 — Supplementary Material 1. [file 12862_2024_2253_MOESM1_ESM.zip › Figure_S2.pdf]

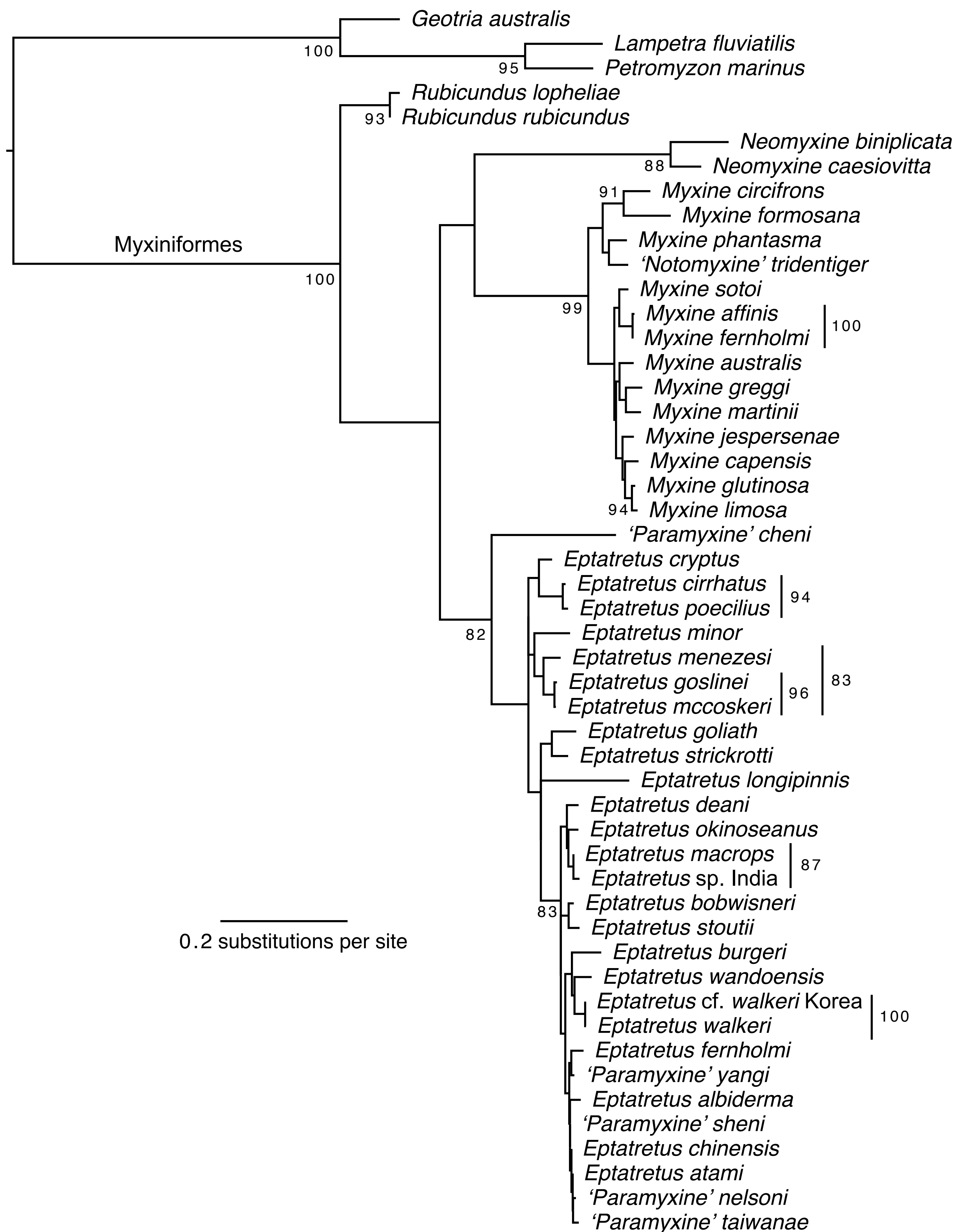

Supplement: Supplementary file 1 — Supplementary Material 1. [file 12862_2024_2253_MOESM1_ESM.zip › Figure_S3.pdf]

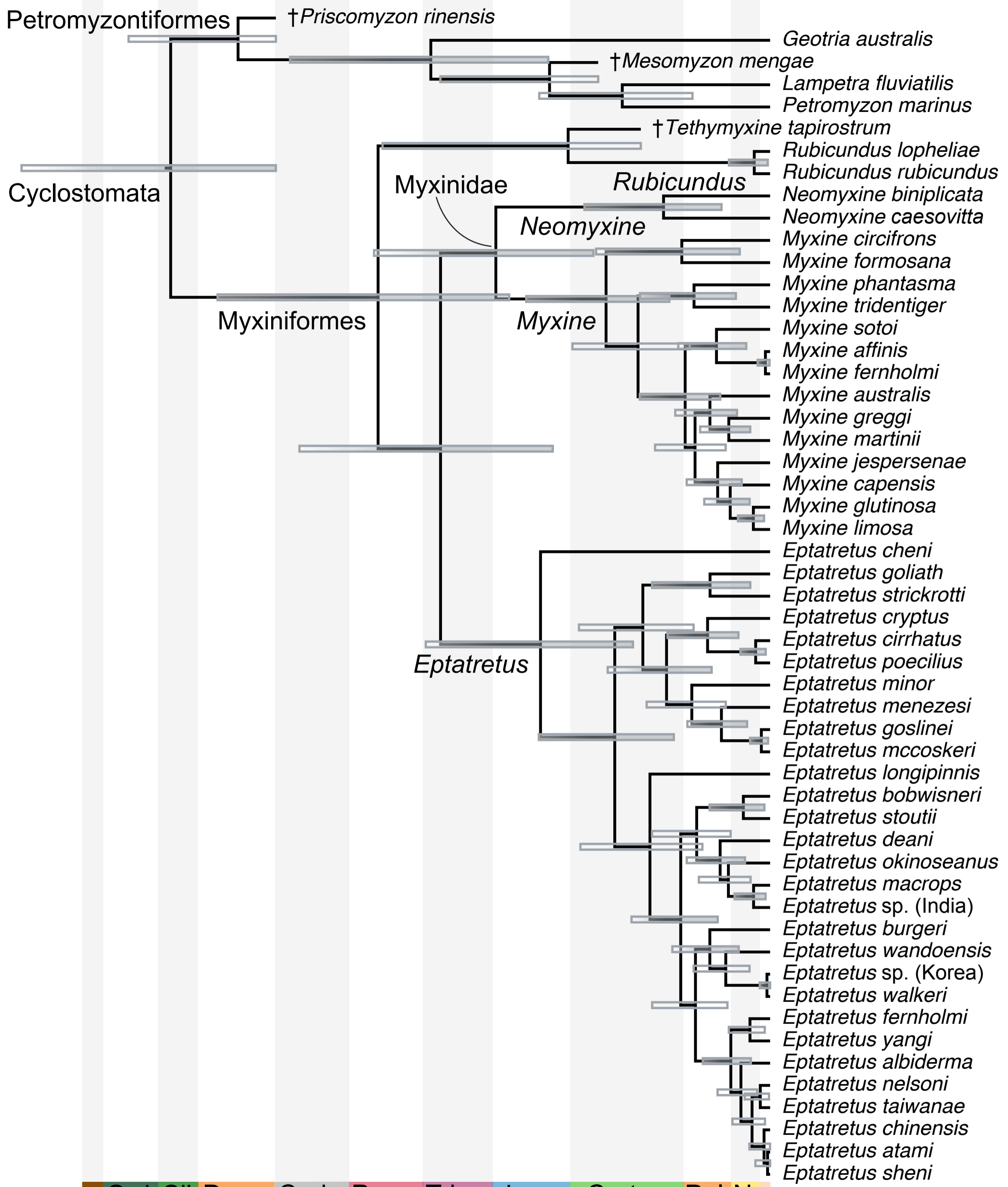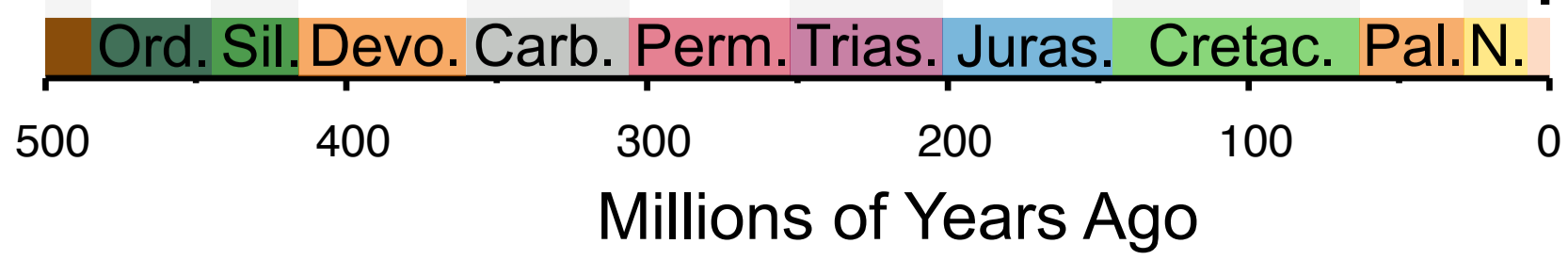

Supplement: Supplementary file 1 — Supplementary Material 1. [file 12862_2024_2253_MOESM1_ESM.zip › Figure_S4.pdf]
